# Supplementary material for: Specificity of end resection pathways for double-strand break regions containing ribonucleotides and base lesions
Source: Nat Commun. 2020 Jun 18;11:3088. doi: 10.1038/s41467-020-16903-4 (PMC7303207; doi:10.1038/s41467-020-16903-4)
Supplement: Supplementary file 1 — Supplementary Information [file 41467_2020_16903_MOESM1_ESM.pdf]

# Supplementary Information

# Specificity of end resection pathways for double-strand break regions containing ribonucleotides and base lesions

**Daley et al.**

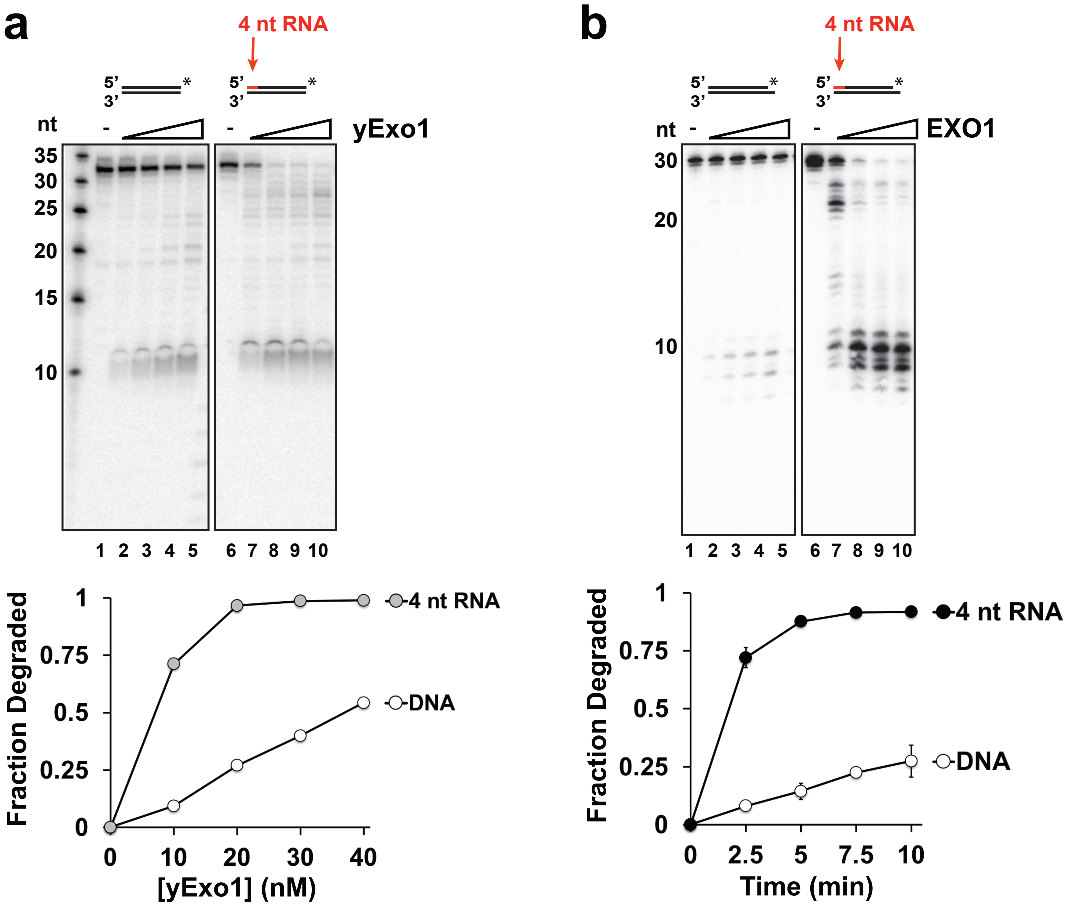


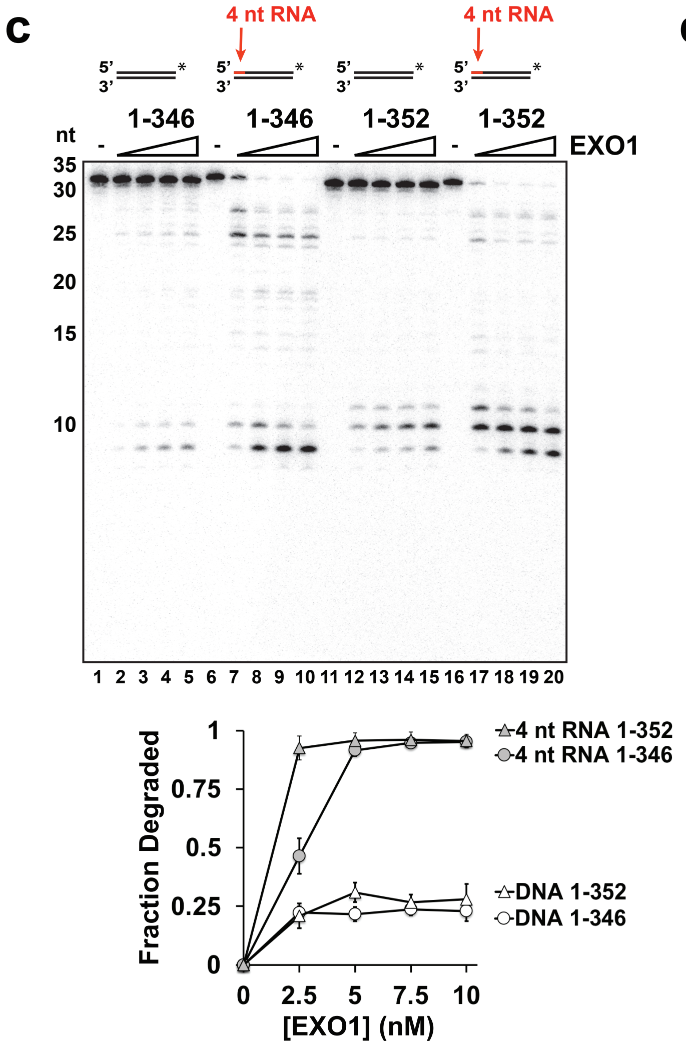


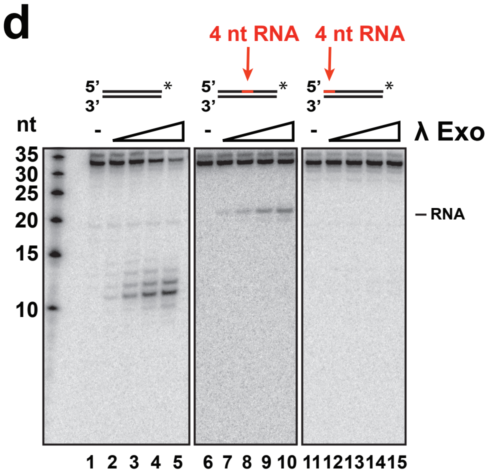


**Supplementary Figure 1: Effect of RNA on EXO1. a**, yExo1 (10, 20, 30, 40 nM) was incubated with the indicated substrate (2.5 nM) at 37˚C for 10 min as in **Figure 1a**. Products were resolved in a 20% denaturing polyacrylamide gel. **b**, EXO1 was incubated with the indicated substrate (2.5 nM) containing 5' overhangs on the unlabeled strand at 37˚C for 10 min as in **Figure 1a**. **c**, EXO1 deletion mutants as indicated (2.5, 5, 7.5, 10 nM) were incubated with the indicated substrate (2.5 nM) at 37˚C for 10 min as in **a**. **d**, Lambda exonuclease (NEB) (0.05, 0.1, 0.2, 03 U) was incubated with the indicated substrate (2.5 nM) at 37˚C for 10 min as in **a**. Error bars in all the panels represent the standard deviation of results from n=3 independent experiments.

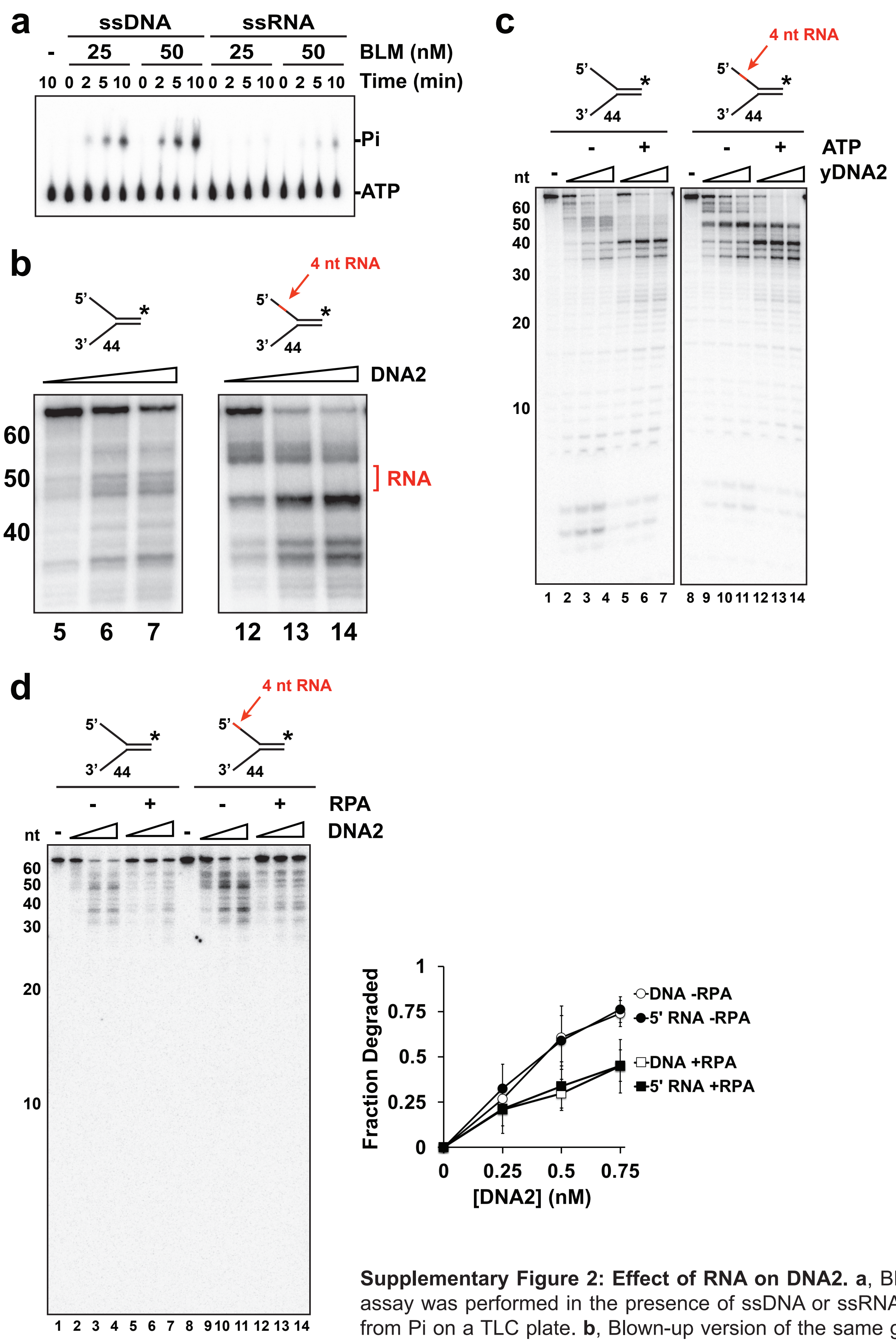


**Supplementary Figure 2: Effect of RNA on DNA2. a**, BLM (25 or 50 nM) ATPase assay was performed in the presence of ssDNA or ssRNA, and ATP was separated from Pi on a TLC plate. **b**, Blown-up version of the same gel shown in **Figure 4a**. **c**, yDna2 (0.25, 0.5, 0.75 nM) was incubated with the indicated Y shaped substrate (2.5 nM) at 30˚C for 5 min in the presence or absence of 2 mM ATP. Reaction mixtures were resolved in a 20% denaturing polyacrylamide gel. **d**, DNA2 (0.25, 0.5, 0.75 nM) was incubated with the indicated Y shaped substrate (2.5 nM) at 37˚C for 5 min in the presence of 2 mM ATP, with or without RPA (10 nM). Error bars in all the panels represent the standard deviation of results from n=3 independent experiments.

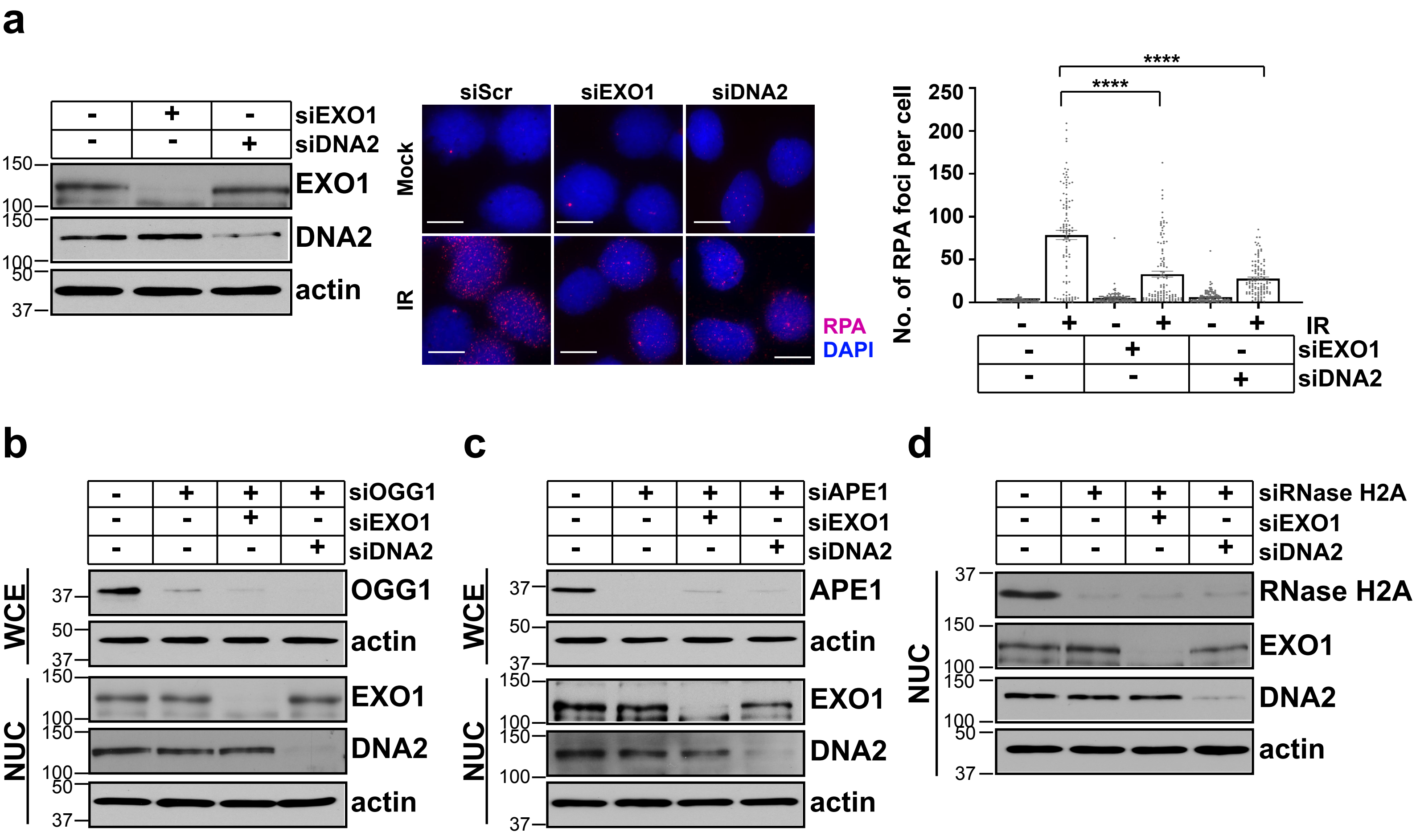


**Supplementary Figure 3: Optimal RPA foci formation depends on both EXO1 and DNA2. a**, U2OS cells were depleted of EXO1 or DNA2 using siRNA as indicated, and knockdown verified by western blotting. Cells were screened for resection defects by quantifying IR-induced RPA foci 3hr after treatment with 6Gy IR. Representative images of mock-irradiated or irradiated (IR) cells immunostained with anti-RPA antibody (red) are shown. Nuclei were stained with 4',6-diamidino-2-phenylindole (blue). Average numbers of RPA foci per nucleus are plotted. All experiments were replicated three times. Error bars depict standard error of the mean and **** indicates $p < 0.0001$; two-tailed unpaired-t-test. The scale bar represents 10 µm for all images. **b**, U2OS cells were depleted of OGG1, EXO1 or DNA2 using siRNA as indicated, and knockdown verified by western blotting. **c**, U2OS cells were depleted of APE1, EXO1 or DNA2 using siRNA as indicated, and knockdown verified by western blotting. **d**, U2OS cells were depleted of RNase H2A, EXO1 or DNA2 using siRNA as indicated, and knockdown verified by western blotting. WCE, whole cell extract; NUC, nuclear extract.

Figure 2a

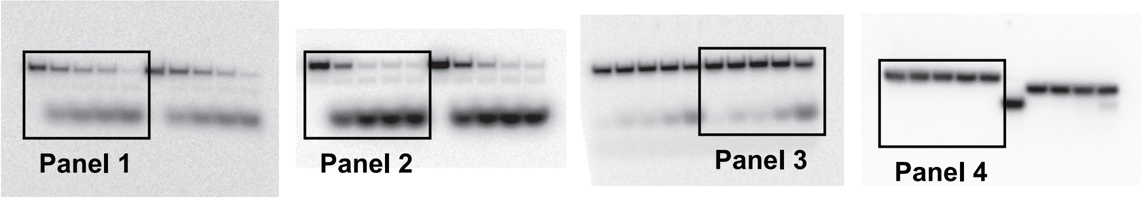


Figure 2b

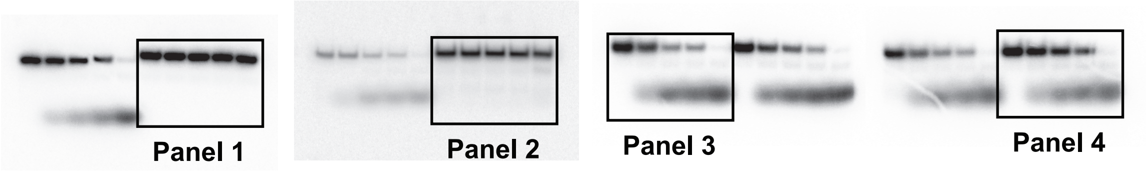


Figure 3b

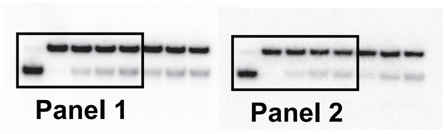


Figure 3c

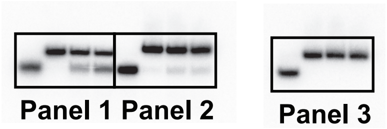


Figure 3d

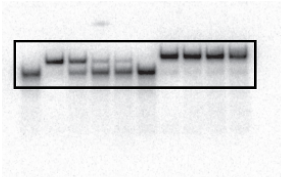

Figure 5c

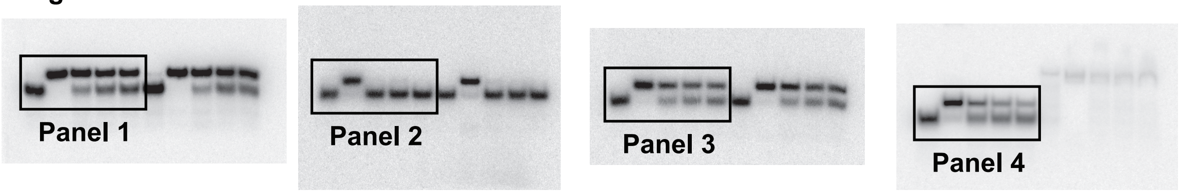


**Supplementary Figure 4: Uncropped gels.** Black rectangles indicate cropping locations.

**Supplementary Figure 3a**

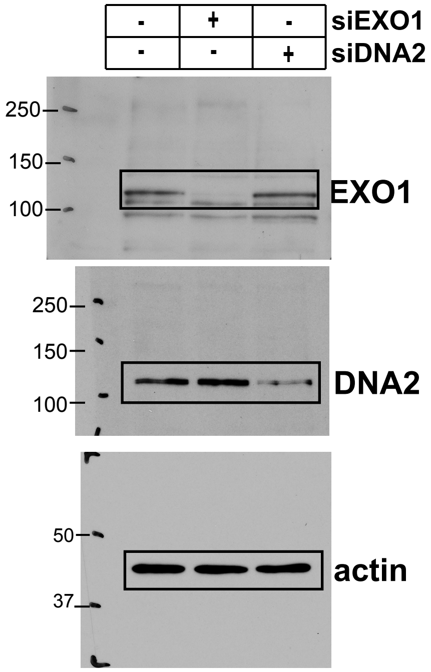


**Supplementary Figure 3b**

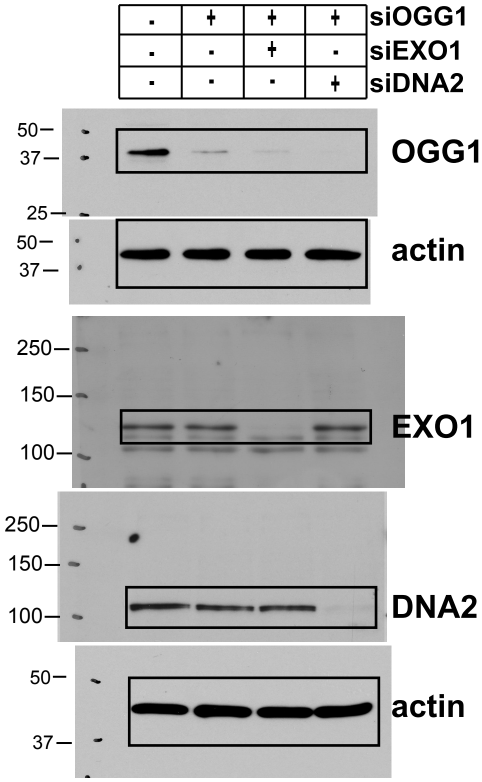


**Supplementary Figure 3c**

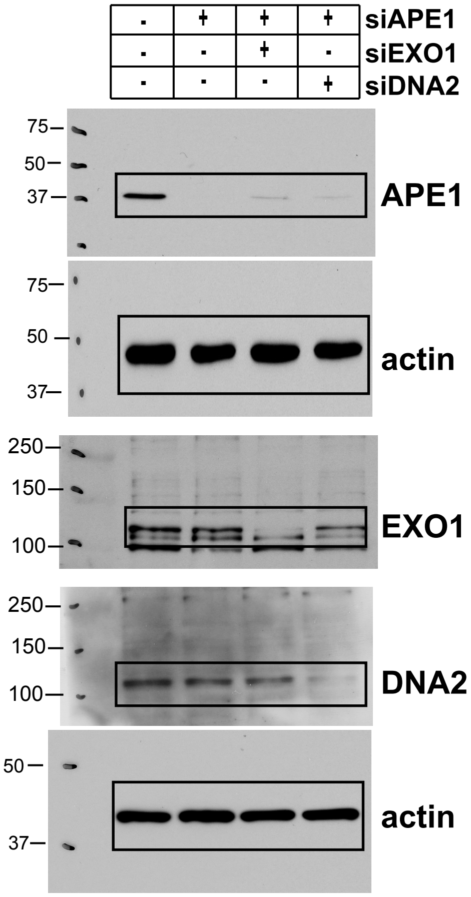


**Supplementary Figure 3d**

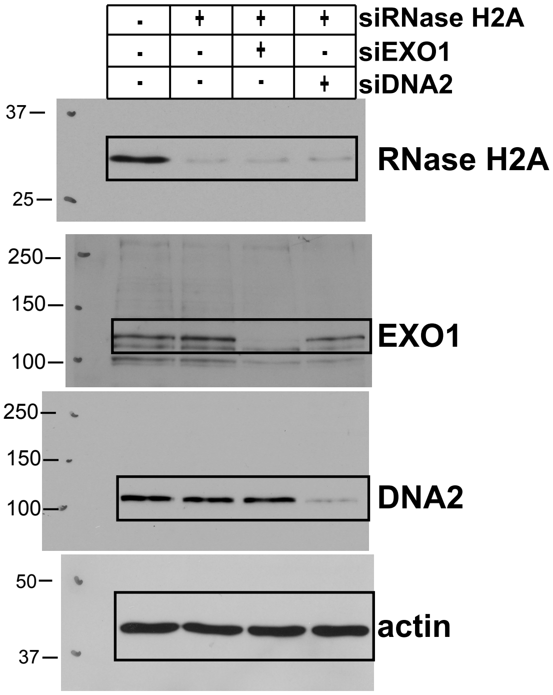


**Supplementary Figure 5: Uncropped western blots.** Black rectangles indicate cropping locations.

**Supplementary Table 1:** Sequences of oligonucleotides used in this study. Ribonucleotides are indicated in red, and alternative bases are shown in bold between parenthesis.

| Name | Sequence | Description |
|---|---|---|
| PSOL9816 | GGACATCTTTGCCCACCTGGTGGTGGGACC | 30 bp dsDNA substrate |
| PSOL9817 | GGTCCCACCACCAGGTGGGCAAAGATGTCC | 30 bp dsDNA substrate |
| PSOL10488 | GGACATCTTTGCCCACCTGGTGGTGGGACC | 1-nt 5' RNA version of PSOL9816 |
| PSOL10208 | GGACATCTTTGCCCACCTGGTGGTGGGACC | 4-nt 5' RNA version of PSOL9816 |
| PSOL11407 | GGACAUCUUUGCCCACCTGGTGGTGGGACC | 10-nt 5' RNA version of PSOL9816 |
| PSOL9275 | GTAAGTGCCGCGGTGCGGGTGCCAGGGCGTGCCCT | Nick substrate (top left) |
| PSOL9276 | TGGGCTCCCCGGGCGCGTACTCCACCTCATGCATC | Nick substrate (top right) |
| PSOL10489 | UGGGCTCCCCGGGCGCGTACTCCACCTCATGCATC | 4-nt 5' RNA version of PSOL9276 |
| PSOL9022 | GATGCATGAGGTGGAGTACGCGCCCGGGGAGCCCAAGGGCACGCCCTGGCACCCGCACCGCGGCACTTAC | Nick substrate (bottom) |
| PSOL9822 | GGACAUCUUUGCCCACCUGGUGGUGGGACC | RNA version of PSOL9816 |
| PSOL9823 | GGUCCCACCACCAGGUGGGCAAAGAUGUCC | RNA version of PSOL9817 |
| PSOL11408 | GGTCCCACCACCAGGTGGGCAAAGATGTCCGATCA | 4-nt 3' tail version of PSOL9817 |
| PSOL11708 | GGACAUCUTTGCCCACCTGGTGGTGGGACC | 4-nt internal RNA version of PSOL9816 |
| PSOL10547 | GGTCCCACCACCAGGUGGGCAAAGAUGUCC | 15-nt 3' RNA version of PSOL9817 |
| PSOL10545 | GGTCCCACCACCAGGTGGGCAAAGATGTCC | 1-nt 3' RNA version of PSOL9817 |
| PSOL10546 | GGTCCCACCACCAGGTGGGCAAAGATGUCC | 4-nt 3' RNA version of PSOL9817 |
| PSOL10207 | GGACATCTTTGCCCACCTGGTGGTGGGACC | 4-nt internal RNA version of PSOL9816 |
| PSOL11408 | GGTCCCACCACCAGGTGGGCAAAGATGTCCGATCA | 5-nt 3' tail version of PSOL9817 |
| PSOL11409 | GGUCCCACCACCAGGUGGGCAAAGAUGUCCGAUCA | RNA version of PSOL11408 |
| PSOL7343 | AGAGGAAAGGAAGAAAGGAGGAAAGGACGTCATAGACGATTACATTGCTAGGACATGCTGTCTAGAGACTATCGC | Y substrate, top |
| PSOL7344 | GCGATAGTCTCTAGACAGCATGTCCTAGCAAGCCAGAATTCGGCAGGCTAGAAAGGAGGAAAGAAGGAAAGGAGA | Y substrate, bottom |
| PSOL10490 | AGAGGAAAGGAAGAAAGGAGGAAAGGACGTCATAGACGATTACATTGCTAGGACATGCTGTCTAGAGACTATCGC | 4-nt internal RNA version of PSOL7343 |
| PSOL10209 | GGACATCTTT**(AP)**CCCACCTGGTGGTGGGACC | AP site version of PSOL9816 |
| PSOL10210 | GGACATCTTT**(8-O-G)**CCCACCTGGTGGTGGGACC | 8-oxo-G site version of PSOL9816 |
| PSOL10486 | GGACATCTTT**A**CCCACCTGGTGGTGGGACC | A-C mismatch version of PSOL9816 |
| PSOL11779 | AAGGTCCCACCACCAGGTGGGCAAAGATGTCC | 2-nt 5' overhang version of PSOL9817 |
| PSOL11711 | AGAGGAAAGGAAGAAAGGAGGAAAGGACGTCATAGACGATTACATTGCTAGGACATGCTGTCTAGAGACTATCGC | 4-nt 5' RNA version of PSOL7343 |

**Supplementary Table 2:** siRNA sequences

| Name | Sequence |
|---|---|
| OGG1 | GAU CAA GUA UGG ACA CUG A |
| APE1 | GCC UUU CGC AAG UUC CUG A |
| RNase H2A | GGA CUU GGA UAC UGA UUA U |
| EXO1 | AG UGU UUC AGG AUC AAC AUC AUC |
| DNA2 | UAC AAC AGC ACU CUU AUU CUG CUC C |

**Supplementary Table 3:** Antibody sources and dilutions

| **Name** | **Vendor** | **Catalog No.** | **Western Blot Dilution** | **Immunofluorescence Dilution** |
|---|---|---|---|---|
| OGG1 | Abcam | ab124741 | 1:1000 | |
| APE1 | Santacruz | sc-17774 | 1:1000 | |
| RNase H2A | Bethyl laboratory | A304-149A | 1:1000 | |
| EXO1 | Bethyl laboratory | A302-639A | 1:1000 | |
| DNA2 | Thermo | PA5-68167 | 1:1000 | |
| Beta-actin | Cell signaling technology | 12262S | 1:3000 | |
| RPA | Millipore Sigma | MABE285 | | 1:2000 |
